# Psychometric Evaluation of the Persian Version of the Successful Aging Inventory

**DOI:** 10.31661/gmj.v9i0.1754

**Published:** 2020-12-18

**Authors:** Masoumeh Fazeli Tarmazdi, Zahra Tagharrobi, Zahra Sooki, Khadijeh Sharifi

**Affiliations:** ^1^Department of Nursing, Trauma Nursing Research Center, Faculty of Nursing and Midwifery, Kashan University of Medical Sciences, Kashan, Iran; ^2^Trauma Nursing Research Center, Faculty of Nursing and Midwifery, Kashan University of Medical Sciences, Kashan, Iran

**Keywords:** Aging, Inventory, Psychometrics Evaluation, Successful Aging

## Abstract

**Background:**

The first step to successful aging planning is to assess the current status using valid instruments. This study aimed to evaluate the psychometric properties of the Persian version of the Successful Aging Inventory (SAI).

**Materials and Methods:**

In the first step, SAI. was translated through forward-backward translation, and its face and content validity were qualitatively and quantitatively assessed. For construct validity assessment, 300 elderly were recruited through multi-stage random sampling. Exploratory factor analysis and known-group comparison were used. SAI reliability through internal consistency and stability was assessed using the Cronbach’s alpha values of the inventory and intraclass correlation coefficient (ICC), respectively. The standard error of measurement, smallest detectable change, and floor and ceiling effects were calculated.

**Results:**

The impact scores, content validity ratios, and content validity indices of all items were more than 1.5, 0.62, and 0.8, respectively. The scale-level content validity index was 0.94. Factor analysis identified four factors for the inventory, which explained 58.17% of the total variance of the SAI score. SAI mean score among mentally healthy participants was significantly higher (P<0.001). The relative frequencies with the lowest and highest possible scores of SAI were 0 and 3.7%, respectively. The Cronbach’s alpha, ICC, standard error of measurement, and the smallest detectable change of SAI were 0.835, 0.999, ±0.47, and 1.9, respectively.

**Conclusion:**

As a valid and reliable instrument, the Persian version of SAI could be used for a successful aging assessment.

## Introduction

Successful aging (SA) is a multidimensional and interdisciplinary context-bound concept [1]. The models and the definitions for this concept are based on two approaches, namely objectivism and subjectivism. The objectivism approach focuses on reducing physical and mental illnesses and disabilities, while the subjectivism model deals with factors such as quality of life, life satisfaction, happiness, and resilience [2]. Previous studies reported that the criteria of SA include absence of illness [3-5], absence of disability [4], normal cognitive and physical functioning [4,5], active engagement in life activities [4], maturation and mastery, positive coping [3], life satisfaction [6], independent life and living environment [3,4], perceived social support [6,7], positive attitude, security, good physical health, and enjoyment in doing activities [7]. Previous studies reported low levels of SA; for instance, a study reported that the rate of SA was 18.6% among Chinese and 25.2% among south Korean older adults [8]. Another study in southern Australia found that the SA rate varied from 11.4% to 87.4% [7]. This rate was also estimated to be 11.9% in the United States [9], 62% in Brazil [10], 21.1% among elderly Danish people, 17% among Swedish and Dutch older adults, 3.1% among Spanish older adults, and 1.6% among Polish older adults [11]. Research in Iran also found that the mean score of SA was 53.2±12.6 and 48.0±13.2 among older women and men (in the possible range of 25–125), respectively, denoting low to moderate levels of SA [12]. SA is affected by many different factors, including personality traits [13], access to support systems, health status, personal abilities, lifestyle [14], financial security, availability of an elderly-friendly environment, and social context [2]. The poor status of SA in the world and Iran and the growing population of older adults highlights the necessity of developing and employing effective plans for SA promotion. The first step to such planning is to assess the current status SA assessment necessitates comprehensive and standard measurement instruments. Currently, there are different SA measurement instruments such as the SA Inventory (SAI) [15-20], Barrett and Murk’s Life Satisfaction Index for the Third Age-Short Form (LSITA-SF) [20,21], Baltes and colleagues’ Selection, Optimization, Compensation (SCO) questionnaire [22], the SA Quiz (SAQ) [23], Robson and et al. SA in the Workplace Index [24], the Korean Elderly SA Scale (KESAS) [25], Goli et al. SA questionnaire [12], and the Multidimensional SA Questionnaire (MSAQ) [2]. Most of these instruments assess only some aspects of SA, such as psychological issues and workplace characteristics. Moreover, some of them include context-bound items and hence, may not be applicable in different contexts. MSAQ [2] and Goli et al. SA questionnaire [12] are Persian SA-specific measurement instruments. Developed based on the findings of a qualitative study, MSAQ is a comprehensive instrument with 54 items in seven subscales, namely psychological well-being, social support, financial and environmental security, physical and mental health, functional health, health-related behaviors, and spirituality. Despite its appropriateness for the Iranian culture [2], many of its items reduce its applicability among older adults. The other Persian SA instrument is the questionnaire developed by Goli *et al*. based on the existing literature and the definition of SA by the World Health Organization. It contains 25 items in the six subscales of health status, social problems, cognitive functioning, financial functioning, spiritual functioning, and mental issues [12]. However, its developers have not yet published it for public use. SAI is one of the specific instruments for SA measurement. Troutman *et al*. developed it in the United States and contained twenty items in five subscales: functional performance mechanisms, intrapsychic factors, gerotranscendence, spirituality, and purposefulness/life satisfaction [15]. The items of this inventory cover the basic dimensions of SA and are appropriate for the Iranian culture. Moreover, because of its small number of items, older adults can respond to it in a short time. Some studies evaluated and confirmed the validity and reliability of SAI. in the United States [15], Korea [16,17], Chile [18], and China [19]. However, it had not yet been validated and adapted for the Iranian culture. Thus, the present study aimed to translate SAI into Persian and evaluate its psychometric properties.

## Materials and Methods

###  1. Participants and Setting

The study population consisted of adults aged 60 years or more who referred to the urban primary healthcare centers in Kashan, Iran, during 2017-2018. Sampling was done through multi-stage sampling. Initially, Kashan city was divided into three hypothetical areas of low, moderate, and good status according to its socioeconomic status. Then, three centers were selected from each area—nine in total. After that, the list of older adults referred to each of the selected centers was created, and a proportionate sample of eligible older adults was selected through simple random sampling. Eligibility criteria were age over 60 years, Iranian nationality, consent for participation, no affliction by known psychosis or mental retardation, no affliction by cognitive problems (determined through a score of more than 20 for the Mini-Mental State Examination scale), ability to communicate in Persian, and ability to hear the voice with or without hearing aids. Exclusion criteria were reluctance to answer study instruments during data collection. The sample size was determined to be 300 based on the rule of thumb recommended by Fawcett and Garity [26].

###  2. Instruments

####  2.1. The Mini-Mental State Examination scale

With ten items, this scale provides an overall estimate of the cognitive state. Its total score can range from 0 to 30, with higher scores showing a better cognitive state [27]. Studies in Iran reported its acceptable validity and reliability with a Cronbach’s alpha of 0.81. Moreover, its sensitivity and specificity at the cutoff score of 21 were reported to be 90% and 84%, respectively [28,29].

####  2.2. Sociodemographic Questionnaire

 This questionnaire included age, gender, marital status, number of children, educational level, and financial status. The qualitative content validity of this questionnaire was approved by six instructors of the Kashan Faculty of Nursing and Midwifery of KAUMS.

####  2.3. General Health Questionnaire (GHQ)

This questionnaire includes 12 items for the assessment of mental health status in the past four weeks [30]. Its items are scored on a four-point 0–3 Likert scale [31], resulting in a possible total score of 0–36. Higher scores of this questionnaire show poorer mental health and vice versa, and the cutoff score for this questionnaire is 14.5. A former study in Iran confirmed its validity and reliability and reported that its Cronbach’s alpha was 0.92 [30].

####  2.4. SAI

This inventory contains 20 items in five subscales. SAI items can be scored through both a dichotomous Yes/No scale and a five-point scale. The points of the dichotomous scale are scored either 0 or 1, resulting in a total score of 0–20. The five-point scale for SAI scoring includes five points, which are scored 0–4, resulting in a total score of 0–80. Higher scores in both scoring systems are interpreted as higher levels of SA. Troutman *et al*. reported that both versions of SAI have acceptable psychometric properties. They found a Kuder-Richardson coefficient of 0.67 for the dichotomously-scored SAI and a Cronbach’s alpha of 0.86 for the SAI version, which is scored on a five-point scale. Thus, the later version has greater internal consistency [15]. In this study, we used the SAI version, which is scored on a five-point scale.

###  3. Data Collection

####  3.1. Primary Stage: SAI Translation 

SAI was translated from English into Persian using the protocol proposed by Wild *et al*. The ten steps of this protocol are preparation, forward translation, reconciliation, back translation, back translation review, harmonization, cognitive debriefing, review of cognitive debriefing results and finalization, proofreading, and final report [32]. Accordingly, necessary permissions for using SAI. were obtained over e-mail from its developers. Two bilingual speakers who were familiar with the literature on gerontology independently translated the English SAI into Persian. Their translations were assessed and merged. Moreover, the item “A relationship with God or some supreme power is important to me” was revised to adapt to the Iranian culture as “Establishing relationships with God and Imams is important to me.” The merged Persian version was given to an expert in Persian literature to evaluate its appropriate wording and grammar. The inventory was revised according to his recommendations. In the next step, a Persian-English translator familiar with the literature on gerontology and healthcare back-translated the Persian version of SAI into English. Then, we assessed the original SAI, its Persian translation, and its back-translated version, resolved disagreements, made necessary revisions and sent the final back-translated English version of the inventory to the developers of its original version. They confirmed our English translation’s conceptual similarity with their original SAI and recommended some revisions made to the final Persian SAI. For cognitive debriefing, the wording appropriateness and comprehensibility of the SAI items were assessed by ten older adults [33] who varied from each other respecting their age, gender, educational level, and socioeconomic status. They were asked about potentially non-comprehensible or offensive items and more comprehensible wording of the items. Necessary revisions were made according to their comments. For instance, the item “I feel able to deal with aging” was revised to “I feel I have been able to face with aging.” The final Persian version of SAI was subjected to psychometric evaluation [32].

####  3.2. First Stage: Content and Face Validity Assessment

For qualitative content validity assessment, ten experts in gerontology, nursing, psychology, and instrument development assessed and commented on the comprehensibility, grammar, wording, adequacy, simplicity, and clarity of the items of the Persian version of SAI. [34]. The quantitative content validity of the Persian version of SAI was assessed by calculating the relaxed content validity ratio (CVRrelaxed) and index (CVI). CVRrelaxed and CVI reflect the essentiality and the relevance of the items, respectively. Accordingly, the same ten experts assessed the essentiality of the items on a three-point Likert scale, and then, their data were used to calculate CVRrelaxed [35,36]. Items with CVRrelaxed values 0.62 or more were considered acceptable [37]. For CVI calculation, the same ten experts rated the relevance of the items on a four-point Likert scale. Their rating scores were used to calculate CVI. According to Waltz and Strickland study, items with CVI values more than 0.79 were considered appropriate [38]. The scale-level CVI (S-CVI) of SAI. was calculated by calculating the average of item CVIs. S-CVI values greater than 0.90 are acceptable [38]. It is noteworthy that the clarity and simplicity of the items were solely assessed in qualitative content validity assessment. For qualitative face validity assessment, the same ten experts assessed any ambiguity or difficulty in understanding the items. Moreover, ten older adults were asked to evaluate ambiguities in the items. After that, the items were revised based on their comments [39]. On the other hand, for quantitative face validity, the same ten experts were invited to rate the importance of items on a five-point Likert scale, and then, their rating scores were used to calculate item impact scores. Items with impact scores more than 1.5 were considered appropriate for further psychometric evaluation [40]. After face and content validity assessment, 300 elderly individuals were asked to fill out the study instruments personally. In the case of limited literacy skills and/or personal preference, the instruments were completed for participants through the personal interview by the first author. The collected data were used to construct validity and reliably assessment.

####  3.3. Second Stage: Construct Validity Assessment and Determining Floor and Ceiling Effects

Construct validity was assessed through exploratory factor analysis and known-group comparison. In factor analysis, factors were extracted through the principal component analysis (with Eigenvalues greater than 1) and scree plot. The minimum acceptable factor loading value was set at 0.5. No common factor loading was observed. For determining floor and ceiling effects, the frequencies of participants who obtained the lowest and the highest possible scores of the Persian version of SAI were calculated [41]. For construct validity assessment through known-group comparison, participants were divided into two groups based on their scores for the GHQ and a cutoff score of 14.5 [34]. Then, these two groups were compared with each other respecting the total mean score of SAI.

####  3.4. Third Stage: Reliability Assessment

The reliability of the Persian version of SAI was assessed using both internal consistency and test-retest stability assessments. For internal consistency assessment, the Cronbach’s alpha values of the inventory and its extracted subscales were calculated. On the other hand, for test-retest stability assessment, twenty older adults (randomly selected from the study sample) re-filled the inventory with a one-week interval. Then, the test-retest intraclass correlation coefficient (ICC) was estimated through the two-way mixed model. Agreement standard error of measurement (SEMagreement) was also calculated, and the smallest detectable change (SDC) was estimated with a confidence level of 95% [42].

###  4. Ethical Considerations

 This study was approved by the Institutional Review Board and the Ethics Committee of Kashan University of Medical Sciences (KAUMS), Kashan, Iran (codes: 2018.3.11.96209 and IR.KAUMS.NUHEPM.REC.139634, respectively). Permissions for conducting the study were obtained from KAUMS and provided to the authorities of the study setting. The research aims were clearly explained to the authorities of the study setting and eligible participants. Elderly individuals who agreed to participate in the study were provided with information about confidential data management and reporting and their freedom to withdraw from the study voluntarily. Finally, written informed consent was obtained from each of them.

###  5. Data Analysis

 Data were analyzed using SPSS 16.0 (SPSS. Inc., IBM., USA). Numerical variables were described through central tendency and dispersion, while categorical variables were described through absolute and relative frequencies. CVR and CVI were calculated for quantitative content validity assessment, and the impact score was calculated for quantitative face validity assessment. The collected data’s appropriateness for the exploratory factor analysis was tested by conducting the Keiser-Meyer-Olkin (KMO) and Bartlett’s tests. The independent-sample t-test was also conducted to compare the known groups, while Cronbach’s alpha and test-retest ICC were calculated for internal consistency and stability assessments, respectively. Floor and ceiling effects were also assessed by calculating the relative frequencies of participants with the lowest and the highest possible total scores of SAI. In all analyses, the level of significance was set at less than 0.05.

## Results

###  1. The Results of Face and Content Validity Assessments

 No changes were made to the items during qualitative face and content validity assessments. The impact scores and the CVRrelaxed values of all items were more than 1.5 and 0.62, respectively. Moreover, the relevance CVI values of all items were 0.8–1, and S-CVI was 0.94.

###  2.The Results of Construct Validity Assessment and Floor and Ceiling Effect Determination

####  2.1. Exploratory Factor Analysis

**Figure 1:**
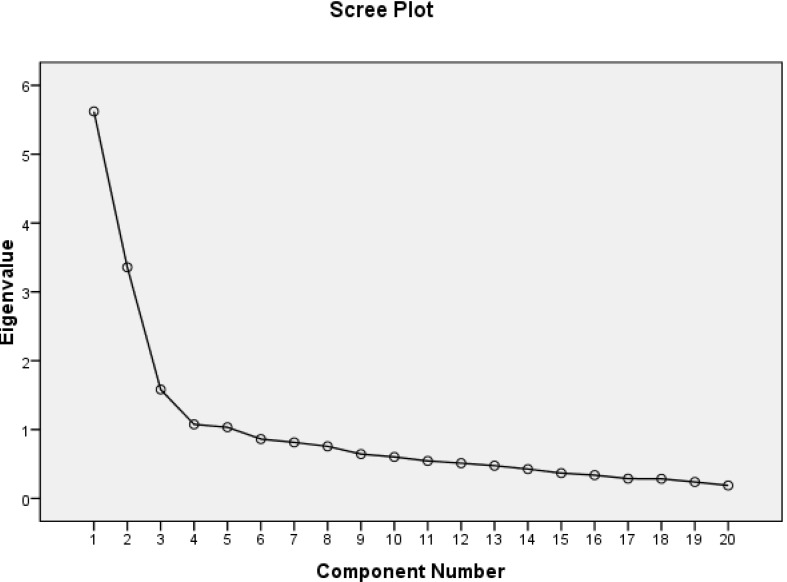
The scree plot of the Persian SIA

 Among 300 participants, 55.7% were female, 82.3% were married, and 30.7% were illiterate. The mean age of participants was 66.75±6.69 years (Table-1). The appropriateness of factor analyses was tested through the KMO and Bartlett’s tests. KMO confirmed sampling adequacy (test value=0.846), and Bartlett’s test revealed that the matrix of inter-correlations among items was appropriate for factor analysis (P<0.001). Subsequently, the exploratory factor analysis resulted in the extraction of four factors with no common factor loading. These factors were labeled positive thinking (eight items), purposeful life (six items), ability to adapt to changes (three items), and wisdom (three items). The Eigenvalues of these factors were 4.637, 3.650, 1.831, and 1.517, respectively. Moreover, these factors respectively explained 23.185%, 18.250%, 9.154%, and 7.583% of the total variance of SAI score. Thus, the total variance explained by these four factors was 58.17%. The scree plots also showed that SAI contained four factors with Eigenvalues more than 1 (Figure-1). The relative frequencies of participants with the lowest and highest possible scores of SAI were 0 and 3.7%, respectively.

####  2.2. Known-Group Comparison

 The mean of participants’ SAI score was 65.446±8.353 (in the possible range of 0–80). Based on the cutoff score of 14.5 for GHQ, participants were divided into two groups, namely mentally healthy and mentally unhealthy. The mean score of SAI among healthy and unhealthy participants was 66.854±7.581 and 62.944±9.084, respectively. The between-group difference was statistically significant (P<0.001).

###  3. The Results of Reliability Assessment

 The Cronbach’s alpha of SAI and its four factors were 0.835, 0.866, 0.829, 0.671, and 0.439, respectively. The test-retest ICC was 0.999 (95% confidence interval: 0.998–1), and SEMagreement and SDC values were 0.47 and 1.9, respectively.

## Discussion

This study sought to translate SAI into Persian and evaluate its psychometric properties. Results showed that the Persian version of SAI has acceptable validity and reliability. During SAI translation, item 15 was modified. Moreover, the ten experts who evaluated SAI content validity recommended some revisions to the inventory to adapt it to the dominant culture and religion in Iran and make it more comprehensible for elderly Iranian people. Such semantic and content modifications are needed for cross-cultural adaptation of instruments [43]. None of the items were modified in qualitative content validity assessment. Moreover, the CVRrelavalues of all items were 0.8–1, which were greater than the minimum critical values presented in Lawshe’s table for ten experts, i.e., 0.62 [37]. Thus, all items were essential. On the other hand, the relevance CVI values of all items were 0.8–1, and S-CVI was 0.94. Item CVI values more than 0.79, and S-CVI values more than 0.90 denote the appropriateness of all items [34,44]. Thus, the content validity of the Persian version of SAI was confirmed. The ten older adults who completed SAI during face validity assessment recommended no revision to its items. Polit *et al*. considered face validity as a criterion for persuading the target population to answer the intended instrument and reported it as a significant factor behind the accuracy of the data obtained from the instrument [34]. Quantitative face validity assessment by ten experts also showed that all items’ impact scores were more than 1.5, confirming the appropriateness of all items [45]. Known-group comparison also showed that mentally healthy participants obtained significantly higher SAI scores than their unhealthy counterparts. In other words, SAI can differentiate mentally healthy older adults from unhealthy ones. This finding confirms that the construct validity of SAI Known-group comparison in an earlier study in Iran also showed that older adults with higher self-reported health status obtained higher scores for all subscales of SA [2]. Internal consistency assessment revealed that the Cronbach’s alpha values of SAI and its positive thinking, purposeful life, ability to adapt to changes, and wisdom subscales were 0.835, 0.866, 0.829, 0.671, and 0.439, respectively. Cronbach’s alpha is an appropriate measure for the assessment of measurement accuracy and internal consistency. Its value can range from 0 to 1 and is interpreted as follows: less than 0.60 indicate week internal consistency; 0.70 show acceptable internal consistency, and 0.80 and more indicate high internal consistency. In other words, Cronbach’s alpha values closer to 1 show greater internal consistency [46]. The Cronbach’s alpha of the original SAI was reported to be 0.86 [15], which is almost the same as the Cronbach’s alpha calculated in the present study. The test-retest intraclass correlation coefficient provides information about the stability and repeatability of the findings of an instrument [47]. Values more than 0.7 are satisfactory, while values more than 0.8 and 0.9 are excellent and optimum, respectively [28]. Therefore, the ICC of the Persian version of SAI, which was 0.999, denotes that it has optimum stability and repeatability and high reliability. Moreover, the SEMagreement value of the Persian version of SAI was 0.47. In other words, the SAI score may change by ±0.47 points in its re-application to the same person. Finally, its SDC was 1.9, with a confidence level of 95%. This study had no significant limitations. Its strengths were multi-stage random sampling, diversity of study participants, and SAI translation through Wild et al. protocol [32].

## Conclusion

 This study revealed that with 20 items in four subscales, the Persian version of SAI has acceptable face, content, and construct validity and acceptable reliability. Its four subscales are positive thinking, purposeful life, ability to adapt to changes, and wisdom. Moreover, it has no floor and ceiling effects, and its SEMagreement was minimal. Hence, it could be used this instrument for SA measurement among elderly Iranian people. Epidemiological studies with confirmatory factor analysis are recommended to provide more information about the psychometric properties of the Persian version of SAI.

## Acknowledgment

 We would like to thank all participating older adults and the Research and Technology Administration of KAUMS, Kashan, Iran. Moreover, we are thankful to Dr. Meredith Troutman for her collaboration.

## Conflict of Interest

 There is no conflict of interest.

**Table 1:** Participants’ Demographic Characteristics

**Characteristics**		**n (%)**
**Gender**	Male	133 (44.3)
	Female	(55.7)167
**Marital status**	Single	(0.3)1
	Married	(82.3)247
	Widowed	(16.3)49
	Divorced	(1)3
**Educational level**	Illiterate	(30.7)92
	Primary	(50)150
	Guidance school	(4.7)14
	Diploma	(11.3)34
	University	(3.3)10
**Children**	Yes	(97.7)293
	No	(2.3)7
**Financial status**	Poor	(19.7)59
	Moderate	(75.3)226
	Good	(5)15
	**Age, y ( Mean±SD )**	66.75±6.69

**Table 2:** SAI Items and Their Factor Loading Values

**No.**	**Item content**	**Factors and factor loading values**			
		**1**	**2**	**3**	**4**
**1**	Ability to perform daily activities			0.578	
**2**	Ability to cope with aging-related changes			0.808	
**3**	Optimism over the future	0.618			
**4**	Ability to face with aging			0.737	
**5**	Ability to cope with life events	0.716			
**6**	Ability to solve problems	0.788			
**7**	Ability to think for solving problems	0.692			
**8**	Enjoyment in doing creative activities				0.568
**9**	Having good feelings	0.779			
**10**	Remembering the loss of significant others		0.664		
**11**	Performing religious activities such as praying		0.746		
**12**	Changing the thinking style in line with aging				0.518
**13**	Intimate relationships with limited number of friends		0.678		
**14**	Thinking about different solutions to problems	0.578			
**15**	Relationships with God and Imams		0.849		
**16**	Thinking about the problems of the next generation				0.787
**17**	Meaningfulness of life		0.645		
**18**	Satisfaction with life	0.738			
**19**	The purposefulness of human creation		0.868		
**20**	Satisfaction with aging at the present moment	0.688			

*The minimum acceptable factor loading was 0.5. Values of less than 0.5 are not presented. **Factor 1: **Positive thinking, with eight items, i.e., items 3, 5–7, 9, 14, 18, and 20. **Factor 2:** Purposeful life, with six items, i.e., items 10, 11, 13, 15, 17, and 19. **Factor 3:** Ability to adapt to changes, with three items, i.e., items 1, 2, and 4.
